# Design of a 5G MIMO Mobile Intelligent Terminal Antenna with Metasurface Loading

**DOI:** 10.3390/s25092927

**Published:** 2025-05-06

**Authors:** He Xia, Heming Fan, Zhulin Liu, Hongxiang Miao, Zhiwei Song

**Affiliations:** 1 State Grid Jibei Electric Power Co., Ltd., Chengde County Power Supply Branch, Bancheng Street Station Road, Chengde 067000, China; 2 State Grid Jibei Electric Power Co., Ltd., Xinglong County Power Supply Branch, Dayoutun Village (Line 112), Xinglong Town, Chengde 067300, China; 3 State Grid Jibei Electric Power Co., Ltd., Fengning County Power Supply Branch, Xinfeng North Road 85#, Dage Town, Chengde 068350, China; 4 School of Electrical Engineering, Xinjiang University, Huarui Street 777#, Shuimogou District, Urumqi 830049, China; suzawer@163.com

**Keywords:** 5G, mobile terminal, MIMO, antenna, miniaturization, metasurface

## Abstract

To achieve multi-band coverage within limited space, reduce antenna types, and enhance communication capabilities, an eight-unit dual-band 5G MIMO antenna array is proposed based on a monopole structure. The antenna operates in two frequency bands (3.23–4.14 GHz and 4.31–5.3 GHz), covering the n78 and n79 bands for 5G applications. The dual-band and miniaturized design of the antenna elements is achieved through the slotting and branch-loading techniques. The orthogonal placement of corner antenna elements is implemented to reduce coupling and optimize spatial utilization, achieving isolation of over 16 dB between elements. The introduction of a metasurface structure further improved isolation by 2 dB and increased the peak gain of the antenna array to 11.95 dBi. A prototype is fabricated and tested, demonstrating the following performance metrics: isolation exceeding 18 dB, gain ranging from 6 to 12 dBi, envelope correlation coefficient below 0.05, channel capacity greater than 41 bps/Hz, diversity gain of approximately 10 dB, total active reflection coefficient below −24 dB, and radiation efficiency exceeding 72%. These results confirm the superior performance of the proposed antenna design.

## 1. Introduction

The design of multi-band MIMO antennas within the limited space of smart mobile terminals faces three core challenges: (1) traditional monopole antennas struggle to achieve dual-band radiation in compact dimensions (typical bandwidth < 15%); (2) strong coupling between densely packed array elements leads to isolation degradation; (3) decoupling techniques often compromise radiation efficiency or spatial utilization (e.g., defected ground structures causing >20% efficiency reduction).

To address these issues, researchers worldwide have proposed various antenna designs [1,2,3]. Compared to 4G, 5G employs multiple antenna elements for simultaneous signal transmission and reception to enable multi-channel data transfer, thereby increasing data capacity and rates (utilizes MIMO technology). The two frequency bands, n78 and n79, are critical for 5G mobile terminals globally (especially in Europe and Asia-Pacific), mainly due to their favorable propagation characteristics and regulatory support [4,5]. However, as the number of antennas increases, coupling effects between MIMO antenna elements become more pronounced, leading to signal interference and system performance degradation [6,7,8]. Consequently, in 5G mobile terminal MIMO antenna decoupling research, numerous studies have proposed various antenna structures and corresponding decoupling methods to improve isolation in compact terminal spaces [9,10,11,12,13,14,15,16,17,18,19,20]. For instance, Ref. [9] presents a compact MIMO antenna for 5G mobile terminals consisting of four closely spaced antenna elements and three lumped components, with a mere 1 mm spacing between adjacent elements. This antenna covers the 3.4–3.6 GHz band with a reflection coefficient below −6 dB and inter-element isolation exceeding 11.6 dB. A broadband, highly integrated four-element MIMO antenna operating from 3.3 to 5.0 GHz with a reflection coefficient below −6 dB and isolation above 10 dB is designed in [10]. In [11], an L-shaped slotted antenna arranges eight identical elements on both sides of a metal frame to form a MIMO array, achieving isolation above 12 dB through optimized element spacing. In [12], an eight-element MIMO array covers 3.5 GHz with high isolation, utilizing balanced slot modes to reduce ground coupling effects and achieving isolation above 17.5 dB with good polarization diversity characteristics. A dual-port MIMO antenna using neutralization line decoupling for wireless USB devices, achieving isolation above 19 dB at 2.4 GHz, is presented in [13]. A self-isolated eight-element MIMO antenna with a T-shaped feed and inverted-L structures printed on both sides of a dielectric substrate, achieving isolation above 20 dB through structural decoupling, is presented in [14]. However, achieving simultaneous miniaturization, dual-band operation, and high isolation in 5G MIMO antennas remains a significant challenge.

To address these challenges, this paper proposes an eight-unit monopole dual-band 5G MIMO antenna design for outdoor smart terminals. First, the slotting and branch-loading techniques are employed to achieve miniaturization and dual-band operation, with relative bandwidths of 25.8% (3.23–4.14 GHz) and 20.7% (4.31–5.3 GHz), surpassing the bandwidth limitations of traditional monopole antennas. Subsequently, a “corner element orthogonal layout + metasurface cooperative decoupling” strategy is proposed for the array architecture. Specifically, edge elements are rotated by 90° to achieve polarization diversity, providing a baseline isolation of 16 dB at 3.5 GHz. Further, a metasurface resonator is introduced to suppress surface wave propagation through electromagnetic localization, achieving peak isolation above 40 dB at 3.5 GHz and increasing the overall antenna gain by 6.6 dBi. The measurement results demonstrate that, within a triangular area of 0.5 × 20 mm × 10 mm per radiating element, the design achieves ECC < 0.05, channel capacity >41 bps/Hz, and radiation efficiency >72% across the entire frequency band. Compared with the previous works in references [9,10,11,12,13,14], this paper addresses several key issues and proposes innovations as follows: 1. Dual-band coverage and miniaturization—a high degree of miniaturization of the antenna elements has been achieved through the use of slot-seam and branch loading techniques to address space constraints. 2. Enhanced isolation and innovative decoupling—a comprehensive strategy of ‘corner orthogonality + metasurface collaborative decoupling’ balances the need for component isolation, bandwidth expansion, and compact layout. 3. Superior overall performance—in addition to isolation and gain improvements, the design also excels in ECC (less than 0.05), diversity gain (about 10 dB), TRAC (less than −24 dB), channel capacity (greater than 41 bps/Hz), and radiation efficiency (more than 72%), fully meeting the stringent requirements of 5G mobile terminals for high data rates, low coupling, and high efficiency.

Additionally, simulations under handheld scenarios confirm radiation stability (TRAC < −24 dB) compliant with IEC 62209−1528 standards, providing a novel antenna solution for high-reliability outdoor communication devices.

## 2. Antenna Design

### 2.1. Antenna Overall Structure

**Figure 1:**
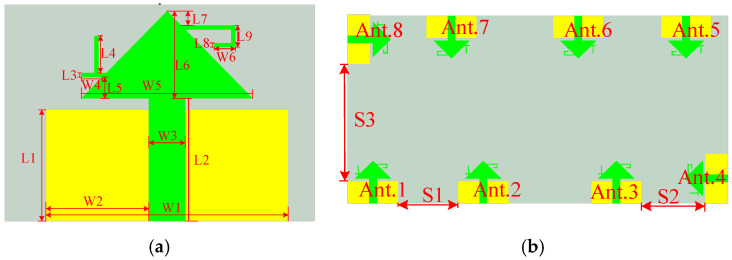
Schematic diagram of antenna unit and array structure: (**a**) antenna unit structure and parameters; (**b**) structure of the 8-unit MIMO antenna array.

**Figure 2:**
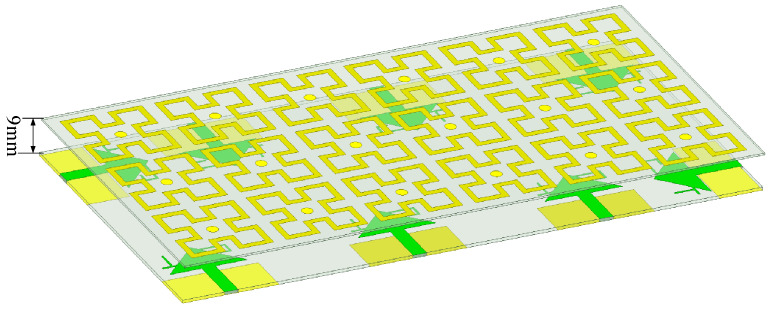
The designed antenna array with metasurface.

Figure 1 shows the unit structure, array layout, and key variables of the proposed 8-unit monopole 5G MIMO antenna (key parameters and their corresponding values are listed in Table 1. As shown, all eight antenna elements share identical structures and dimensions, printed on a 0.8 mm thick FR4 dielectric substrate (*ε*_r_ = 4.4, tanδ = 0.02), with a cleared ground plane. The primary radiating surface of each monopole antenna element is an isosceles triangle with an area of 0.5 × 20 mm × 10 mm (0.5 × 0.226 *λ*_0_ × 0.112 *λ*_0_), connected to a 2 mm wide microstrip line. To ensure coverage of the n78 and n79 bands for 5G applications in smart mobile terminals, the proposed 8-element MIMO antenna is designed for dual-band operation, covering 3.3–4.07 GHz and 4.21–5.2 GHz. To reduce inter-element coupling, the ground plane is cleared, and the spatial arrangement of the ground plane and antenna elements is optimized to achieve isolation greater than 16 dB between the elements. To further enhance isolation, a metasurface is introduced 9 mm above the cleared 8-element MIMO antenna for decoupling, as depicted in Figure 2. (When making the prototype, the materials and equipment for making the antenna were provided and completed by Shenzhen Yuchi Technology Co., Ltd., Shenzhen, China).

In our work, S-parameters (scattering parameters) are employed to describe the reflection and transmission characteristics of the antenna network. The detailed explanation is as follows:

1. Basic Definition of S-Parameters: For a multi-port network (e.g., a two-port system), let the amplitudes of the incident and reflected waves be represented by aaa and bbb, respectively. For any port iii, the S-parameter is defined as follows:

(1) $$S_{ij}=\frac{b_{i}}{aj}(i\neq j)$$

where, $S_{ii}$ (e.g., S_11_ or S_22_) is the reflection coefficient at port *i*, and $S_{ij}$ (i≠j) represents the transmission coefficient from port *j* to port *i*.

2. Reflection Coefficient S_11_: When all the other ports are terminated with a matching load of characteristic impedance Z_0_ (typically 50 Ω), the reflection coefficient at port 1 is given by the following:

(2) $$S_{11}=\frac{b_{1}}{a_{1}}$$

In practice, S_11_ is often related to the antenna input impedance Z_in_ and the characteristic impedance Z_0_ via the following formula:

(3) $$S_{11}=\frac{Z_{in}-Z_{0}}{Z_{in}+Z_{0}}$$

S_11_ is usually expressed in decibels (dB) as follows:

(4) $$S_{11}(dB)=20{log}_{10}\left|S_{11}\right|$$

This parameter indicates how well the antenna is matched—lower S_11_ values signify better matching and lower reflection losses.

3. Transmission Coefficient S_21_: S_21_ represents the transmission characteristics from port 1 to port 2, defined as follows:

(5) $$S_{21}=\frac{b_{2}}{a_{1}}$$

It reflects the coupling or isolation between two antenna elements. A lower S_21_ value indicates higher isolation and reduced mutual interference between the antenna elements.

4. Simulation Principle: In full-wave electromagnetic simulation software (e.g., HFSS), Maxwell’s equations are numerically solved to obtain the scattering properties of the structure, from which the S-parameters are extracted. These parameters quantify how the antenna performs in terms of impedance matching (through S_11_) and inter-element coupling (through S_21_) across the operating frequency range, thereby guiding design optimization.

### 2.2. Antenna Unit Design Process

**Figure 3:**
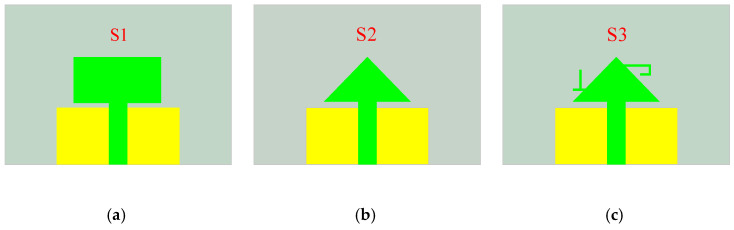
The evolution process of antenna unit structure: (**a**) S1; (**b**) S2; (**c**) S3.

**Figure 4:**
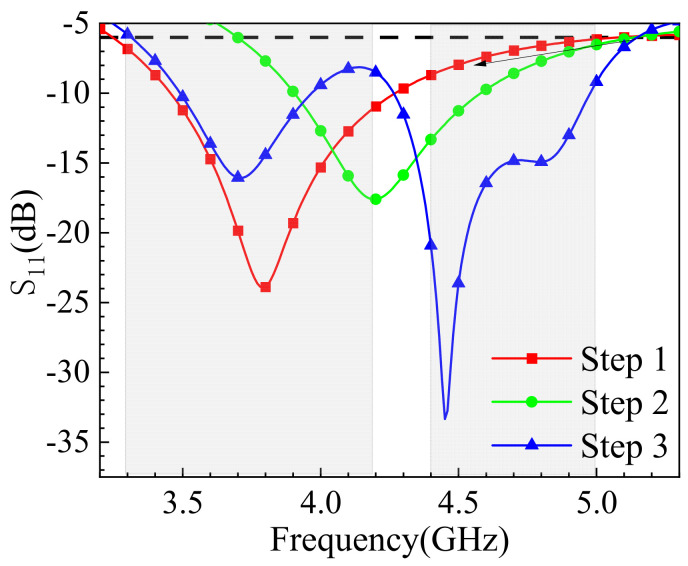
Comparison of simulation results of S_11_ parameters during the evolution process of antenna units.

The design process of the antenna element can be divided into three steps, as illustrated in Figure 3, which shows the evolution of the monopole antenna element, and Figure 4, which presents the corresponding S_11_ simulation results. In Stage 1 (S1), the antenna exhibits poor impedance matching, particularly in the n79 band, failing to meet the design objectives. To achieve the miniaturization of the antenna element, the rectangular radiating element is modified with corner truncations, as shown in Stage 2 (S2). It is observed that the resonant frequency shifts to the right, and the bandwidth narrows due to the reduced size. To meet the design goals, two branches are added to the isosceles triangular radiating patch (as shown in Stage 3 (S3)) to extend the current path and achieve dual-band characteristics. As indicated by the blue curve in Figure 4, the antenna element successfully achieves dual-band operation, effectively covering the n78 and n79 bands with excellent impedance matching.

### 2.3. MIMO Antenna Array Evolution and Simulation Analysis

**Figure 5:**
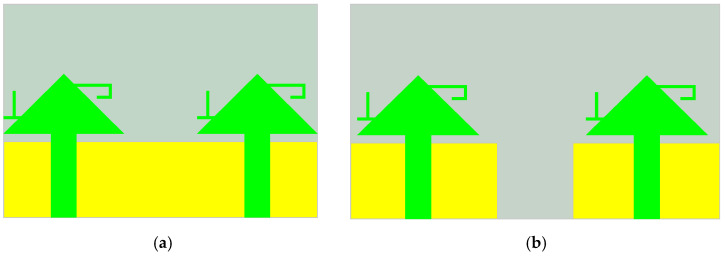
Structure before and after antenna ground plane slotting treatment: (**a**) before ground clearing treatment; (**b**) after ground clearing treatment.

**Figure 6:**
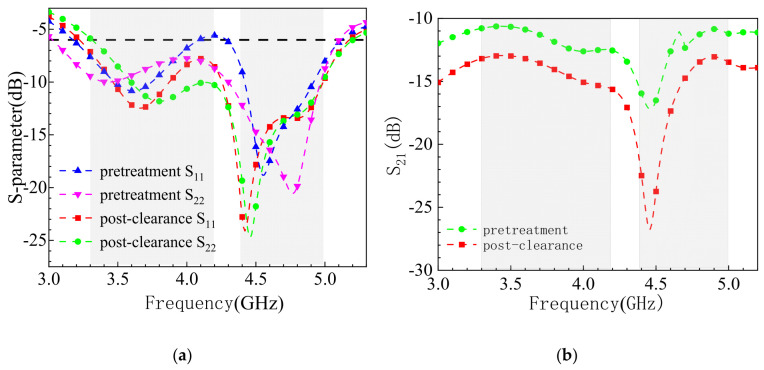
Comparison of S-parameter simulation results before and after ground plane slotting treatment: (**a**) reflection coefficient; (**b**) coupling.

Before analyzing the eight-element array, a two-element structure is first examined, as shown in Figure 5. To improve the isolation between antenna elements, the ground plane is cleared, and the reflection coefficients of the antenna are depicted in Figure 6. By modifying the ground plane structure through clearing, the distribution of the electromagnetic field is adjusted, reducing the propagation of electromagnetic waves on the ground plane and thereby decreasing mutual coupling between antenna elements. As shown in Figure 6b, at the first resonant frequency of 3.5 GHz, the isolation between antenna elements before clearing is greater than 10 dB, while after clearing, the isolation improves by 3 dB, reaching over 13 dB. At the second resonant frequency of 4.5 GHz, the isolation before clearing is greater than 15 dB, and after clearing, it increases to 25 dB. Thus, ground plane clearing significantly enhances isolation at the second resonant frequency, with the next focus on decoupling at the first resonant frequency of 3.5 GHz. Figure 6a shows that the reflection coefficient remains largely unchanged before and after clearing, demonstrating that ground plane clearing has minimal impact on the antenna bandwidth. The primary cause of coupling in MIMO antennas is the spatial distance between antenna elements, leading to the mutual interference of radiated electromagnetic waves. Adjusting the distance between antenna elements can effectively reduce coupling and improve isolation. However, due to the size constraints of mobile devices, the distance between the four groups of antenna elements arranged in parallel on both sides of the substrate cannot be arbitrarily adjusted, making it impossible to achieve ideal decoupling solely through spacing adjustments.

**Figure 7:**
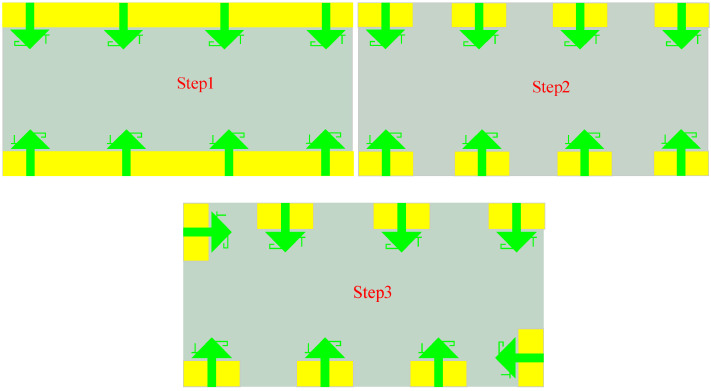
Spatial layout variation process of eight-cell MIMO antennas.

**Figure 8:**
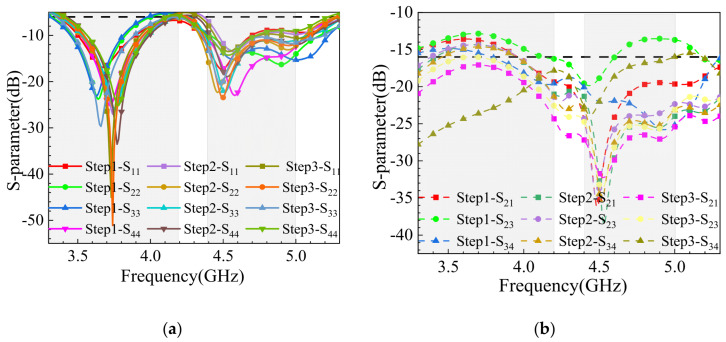
Comparison of S-parameter simulation results for spatial layout change process: (**a**) reflection coefficient; (**b**) coupling degree.

**Figure 9:**
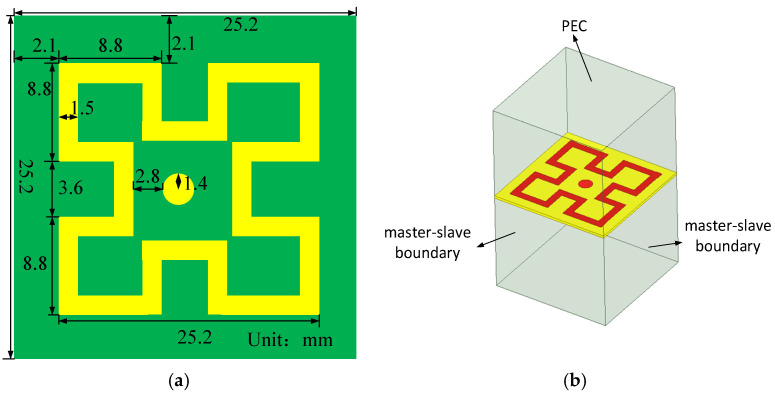
Metasurface diagram: (**a**) metasurface structure diagram; (**b**) simulation setup diagram.

The evolution of the antenna array layout is divided into three steps, as shown in Figure 7, with the corresponding reflection coefficients and coupling simulation results depicted in Figure 8. It is evident that the array layout exhibits symmetry, allowing the analysis of one side’s four elements. Orthogonal polarization, achieved by aligning the polarization directions of two antenna elements orthogonally, reduces coupling between elements and is less dependent on spatial distance. Thus, reorganizing the spatial layout of the 8-element MIMO antenna can improve isolation between elements. After reconfiguring the MIMO antenna structure using ground plane slotting and orthogonal polarization techniques, the reflection coefficients of the antenna elements show minimal changes across the three steps. As shown in Figure 8a, the MIMO array covers the frequency bands of 3.3–4.2 GHz and 4.4–5.3 GHz. Figure 9b demonstrates that without additional decoupling techniques, the isolation between antenna elements meets the international standard of above 10 dB but is not ideal. The use of ground plane clearing (Step 2) enhances isolation. Particularly in Step 3, the horizontal placement of Ant.4 ensures orthogonal radiation directions between Ant.3 and Ant.4, achieving a peak isolation of 27 dB at 3.3 GHz, with the overall MIMO antenna isolation exceeding 16 dB.

To further enhance isolation, metasurface technology is introduced. Metasurface technology involves designing periodic structures to suppress or reflect surface electromagnetic waves at specific frequency bands, thereby reducing coupling, increasing bandwidth, and improving gain.

The unit cell of the metasurface periodic structure is shown in Figure 9a. To obtain the parametric characteristics of the metasurface, the top and bottom boundaries are set as perfect electric conductor (PEC) conditions in the HFSS (HFSS 2021) 3D electromagnetic simulation software, while the four sides are configured with two sets of periodic master–slave boundaries. The bottom substrate is selected as F4b with a thickness of 0.508 mm (relative permittivity *ε*_r_ = 2.2; dielectric loss tangent tanδ = 0.007), as illustrated in Figure 9b.

**Figure 10:**
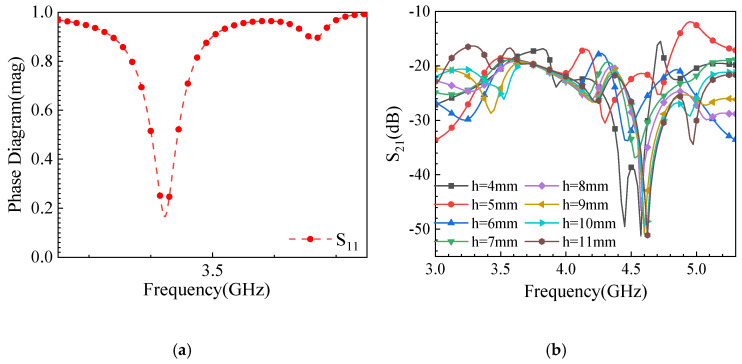
Metasurface simulation results: (**a**) phase map of the metasurface; (**b**) the influence of height variation in metasurface installation on isolation.

Figure 10a shows the phase simulation results of the metasurface unit cell, indicating resonance in the frequency range of 3.53-3.58 GHz. This confirms that the unit cell can absorb or reflect electromagnetic waves in this band to achieve coupling reduction. Subsequently, the periodic unit cells are arranged in an array and integrated with the antenna model. After conducting a parametric sweep of the metasurface height (h) from 4 mm to 11 mm (see Figure 10), we observed that h = 9 mm yields the best isolation performance around 3.5 GHz (approximately −40 dB) and generally provides higher isolation across the 3.3–5.0 GHz range compared to other heights. This occurs because, at 9 mm, the metasurface effectively suppresses surface waves while maintaining an enhanced main beam, striking an optimal balance between isolation improvement and overall radiation performance. Furthermore, from a manufacturing and mechanical standpoint, 9 mm is a practical choice for integration into a mobile terminal. Therefore, we selected h = 9 mm as the optimal metasurface height to maximize isolation while accommodating real-world design constraints. Figure 10b demonstrates the effect of varying the height (h) of the metasurface unit on isolation, with the optimal performance observed at h = 9 mm.

**Figure 11:**
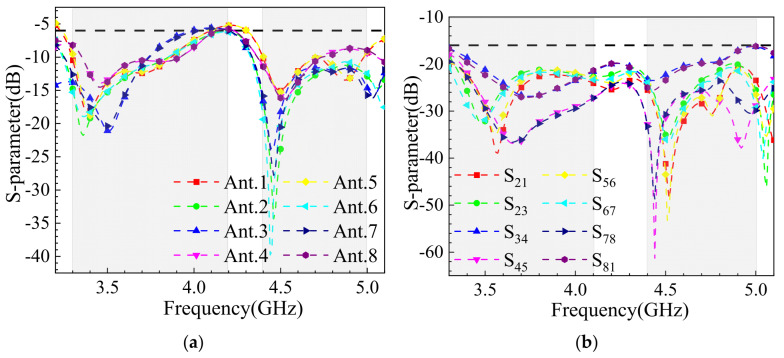
S-parameter simulation results of loaded metasurface: (**a**) reflection coefficient; (**b**) coupling.

The simulated S-parameters of the proposed 8-element MIMO array antenna are shown in Figure 11. It is evident that the array’s impedance bandwidth effectively covers the N78 and N79 frequency bands, with the overall isolation between antenna elements exceeding 18 dB. As shown in Figure 11b, after loading the periodic metasurface, the isolation at 3.5 GHz exhibits a significant dip, indicating that the metasurface effectively absorbs and suppresses electromagnetic wave propagation at this frequency, thereby reducing mutual interference between antenna elements. At 3.5 GHz, the peak isolation between antenna elements reaches 40 dB, demonstrating the effectiveness of the metasurface in enhancing decoupling performance.

**Figure 12:**
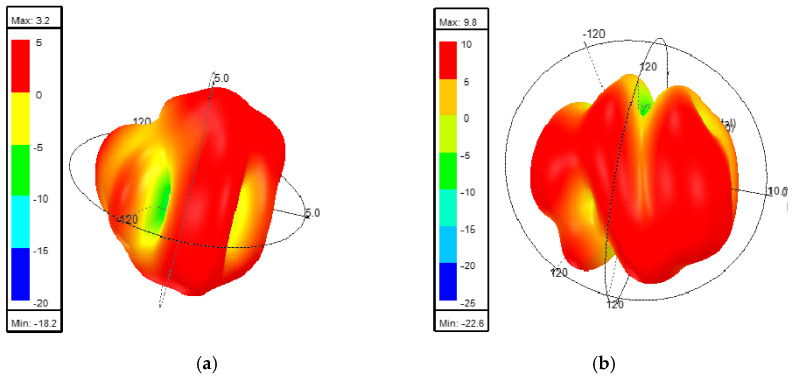
The 3.5 GHz gain comparison before and after loading the metasurface: (**a**) without metasurface; (**b**) with metasurface.

In addition, the introduction of the metasurface structure significantly impacts the gain of the MIMO antenna. Taking 3.5 GHz as an example, the 3D gain patterns of the MIMO antenna before and after loading the metasurface are simulated, as shown in Figure 12a,b. From Figure 12a, it can be observed that the maximum gain of the MIMO antenna before loading the metasurface is 5.35 dBi. In contrast, Figure 12b demonstrates that the maximum gain of the MIMO antenna after loading the metasurface increases to 11.95 dBi. This represents a gain improvement of 6.6 dBi, highlighting the significant enhancement in radiation performance achieved by incorporating the metasurface structure.

## 3. MIMO Array Performance Evaluation and Environmental Adaptation Analysis

To evaluate the performance of the proposed 8-element MIMO antenna, its diversity performance is thoroughly investigated by analyzing key metrics such as envelope correlation coefficient (ECC), total active reflection coefficient (TARC), diversity gain (DG), and channel capacity. Since Ant.1 to Ant.4 and Ant.5 to Ant.8 are printed on opposite sides of the smart terminal model, their spatial separation minimizes mutual influence. Additionally, due to the symmetrical arrangement of the antenna elements on both sides, the data for Ant.1 to Ant.4 and Ant.5 to Ant.8 are nearly identical. To avoid clutter, only the data for Ant.1 to Ant.4 are included in the charts.

**Figure 13:**
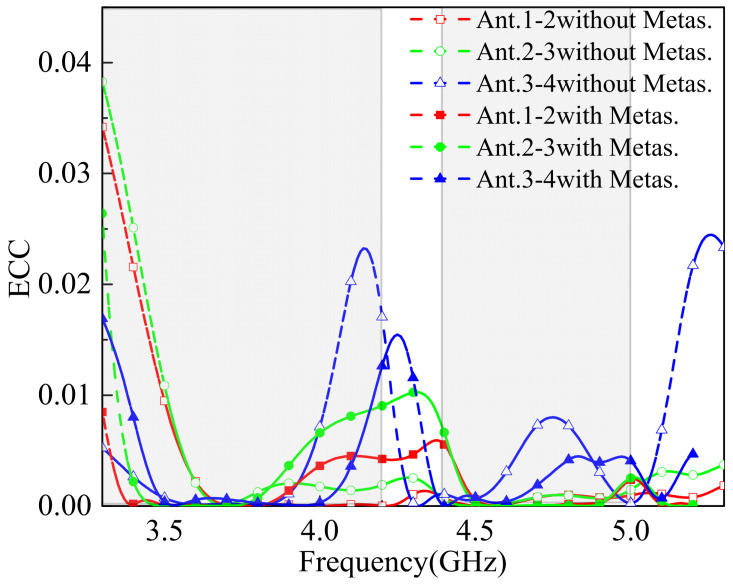
The simulated ECC.

The ECC value is typically related to the coupling level between antenna elements. According to the design standards for MIMO antennas in terminal devices, the ECC should be less than 0.5. The simulated ECC results, as shown in Figure 13, indicate that all the ECC values are below 0.05. Across the entire operating band, the highest ECC value occurs between Ant.2 and Ant.3, yet it remains below 0.04. This demonstrates excellent diversity performance and low coupling, meeting the stringent requirements for MIMO antenna applications in 5G mobile terminals.

**Figure 14:**
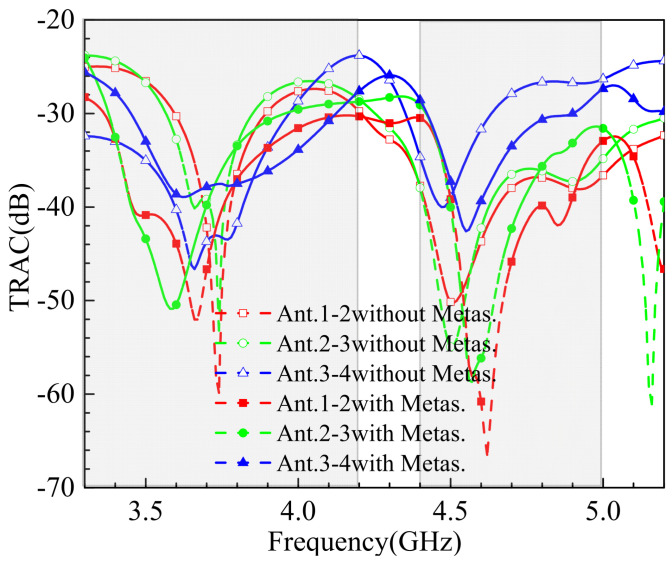
The simulated TRAC.

The total active reflection coefficient (TRAC) is a critical metric for evaluating the performance of MIMO antenna systems. It represents the square root of the ratio of the total reflected power to the total incident power. A lower TRAC value indicates higher efficiency in the antenna’s reception of all incident power. For a dual-port MIMO system, the TRAC value can be calculated using Equation (6). The TRAC of the proposed antenna is below −24 dB, as shown in the simulated results in Figure 14. This demonstrates the antenna’s excellent efficiency in power reception and its suitability for high-performance MIMO applications in 5G mobile terminals.

(6) $$TARC=\frac{\sqrt{{\left|\left(S_{11}+S_{12}\right)\right|}^{2}+{\left|\left(S_{21}+S_{22}\right)\right|}^{2}}}{\sqrt{2}}$$

**Figure 15:**
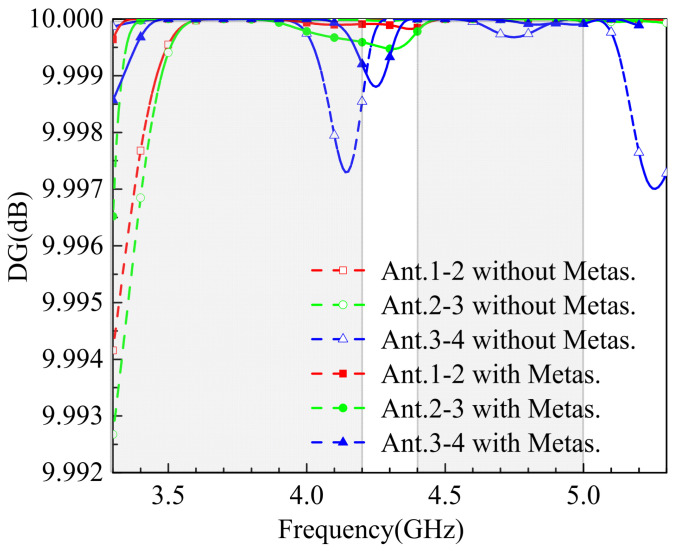
The simulated DG.

Diversity gain (DG) represents the ability to improve signal quality in multipath propagation environments by leveraging the differences between multiple antennas. This gain effectively reduces signal fading and interference, thereby significantly enhancing the reliability and performance of the communication system. The DG value is calculated using Equation (7). The designed MIMO antenna achieves a DG value ranging from 9.92 to 10 dB, as shown in the simulation results in Figure 15. This high DG value underscores the antenna’s robust performance in mitigating multipath effects and ensuring reliable communication in diverse environments.

(7) $$DG=DG_{0}\sqrt{1-\rho_{e}}$$

**Figure 16:**
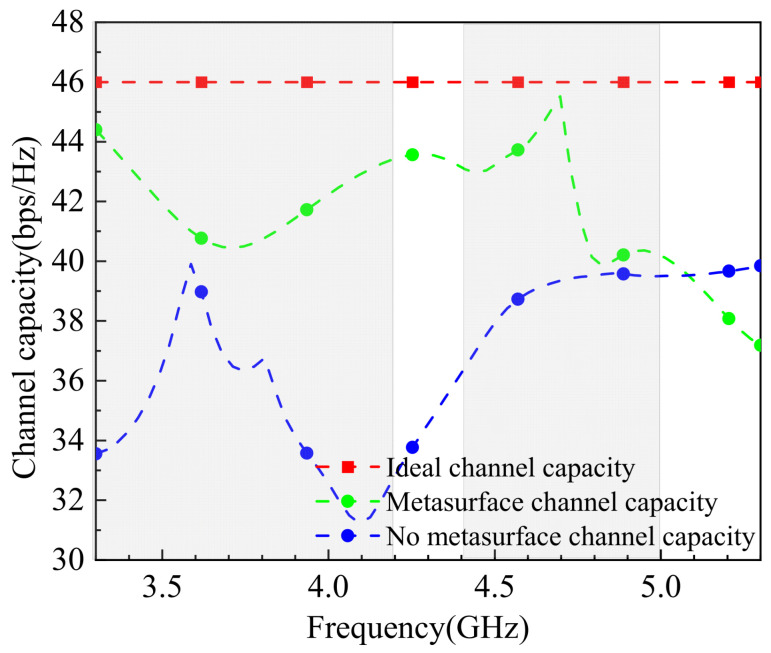
The simulated channel capacity.

In MIMO antenna systems, channel capacity refers to the maximum amount of data that can be transmitted per unit time by leveraging the characteristics of multiple antennas and multipath propagation environments. The channel capacity is calculated using Equation (8), and the simulation results are shown in Figure 16. For the calculation, it is assumed that the transmit antennas are uncorrelated, and the average channel capacity is derived from 100,000 independent and identically distributed Rayleigh fading instances, with a signal–noise ratio (SNR) of 20 dB. The results indicate that the 8-element MIMO antenna achieves a channel capacity ranging between 39.5 and 44 bps/Hz in the 5G Sub−6 GHz bands (primarily 3.3–3.6 GHz and 4.8–5.0 GHz). Compared to the ideal 8-element MIMO antenna channel capacity, the tested antenna’s capacity is slightly lower than the ideal peak. The significant oscillations observed in the 3.3–4.1 GHz range may be attributed to computational errors due to the large bandwidth and extensive data. Nevertheless, the results demonstrate that the designed 8-element MIMO antenna exhibits excellent spatial multiplexing performance.

(8) $$C=log10_{2}\det\left(I_{M}+\frac{\rho_{T}}{M}HH^{+}\right)$$

Table 2 provides a comparison of the proposed 8-element MIMO antenna with other MIMO antennas reported in the literature. From the table, it is evident that the proposed antenna exhibits superior overall performance compared to those in [15,16,17,18], particularly in terms of isolation, bandwidth, and compact size. While the antenna efficiency is slightly lower than that of [17], the ECC values are better than those of [17,18]. The isolation performance of the proposed antenna surpasses the optimal values reported in other references, exceeding 18 dB.

Overall, the proposed antenna demonstrates significant advantages over the referenced designs, making it a competitive candidate for 5G MIMO applications in mobile terminals.

**Figure 17:**
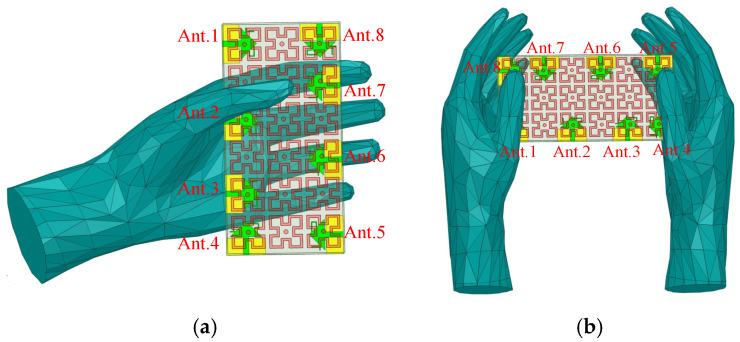
Handheld terminal method: (**a**) one-handed use mode; (**b**) two-handed use mode.

Impact of Application Scenarios on Antenna Performance: Since the designed 8-element MIMO antenna is primarily intended for 5G mobile devices, it is essential to evaluate the impact of real-world user interactions on its performance. Using the HFSS electromagnetic simulation software, the antenna model’s performance is tested under different handheld scenarios. Typically, handheld terminal usage can be categorized into single-hand and dual-hand modes. Figure 17a,b illustrate the single-hand and dual-hand usage modes, respectively, for the antenna model. These simulations help assess the antenna’s robustness and performance degradation in practical usage scenarios, ensuring its reliability in real-world applications.

**Figure 18:**
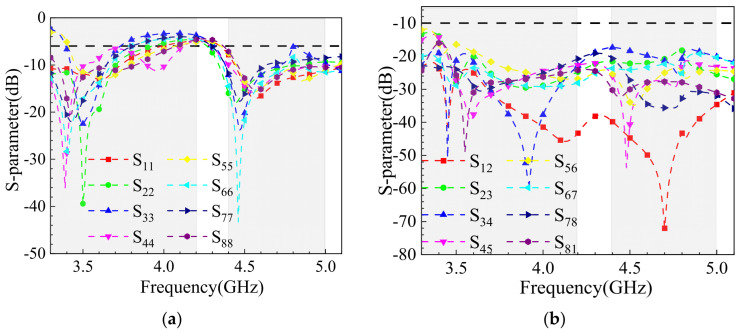
S-parameter simulation results for one-handed use mode: (**a**) reflection coefficient; (**b**) coupling.

As shown in Figure 18a, in single-hand usage mode, the thumb obstructs Ant.2, while the palm model partially blocks the bottom of the remaining antenna elements (except Ant.1, Ant.4, Ant.5, and Ant.8). The varying degrees of obstruction caused by the palm model result in the absorption of spatially propagating electromagnetic waves by hand tissues, affecting the antenna elements to different extents. Figure 18a,b present the simulated reflection coefficients and coupling levels of the MIMO antenna in single-hand mode. The impact of single-hand usage on the reflection coefficients is minimal, with the antenna bandwidth still covering 3.3–3.6 GHz and 4.8–5.0 GHz while maintaining reflection coefficients below −6 dB. Near 3.3 GHz, the isolation of some antenna elements is significantly affected, with the lowest isolation reaching 12 dB. However, within the operating frequency range, the isolation between antenna elements remains above 10 dB, meeting the MIMO antenna standards. This demonstrates the antenna’s robustness in practical single-hand usage scenarios.

**Figure 19:**
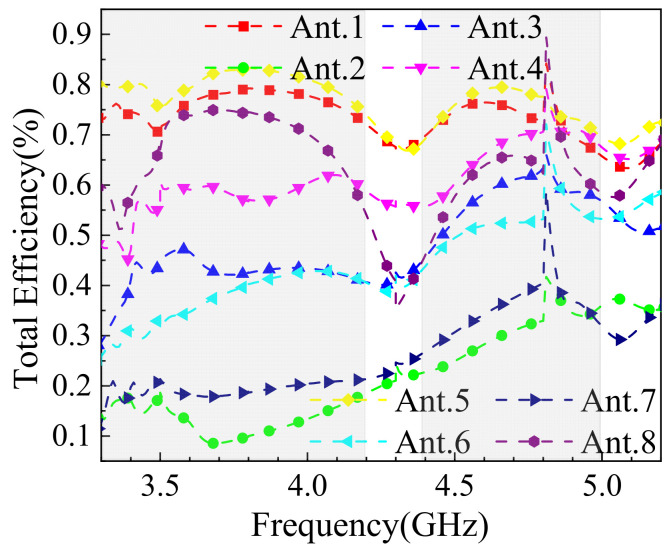
Simulation results of antenna efficiency for one-handed use mode.

Figure 19 shows the simulated radiation efficiency of the MIMO antenna in single-hand mode. The efficiency of the obstructed antenna elements is generally lower, particularly at 3.5 GHz, where Ant.2 exhibits an efficiency of only 15%. However, the unobstructed elements maintain efficiencies above 45%, ensuring the mobile device’s normal operation. This highlights the antenna’s ability to maintain functionality despite partial obstruction in real-world usage scenarios.

**Figure 20:**
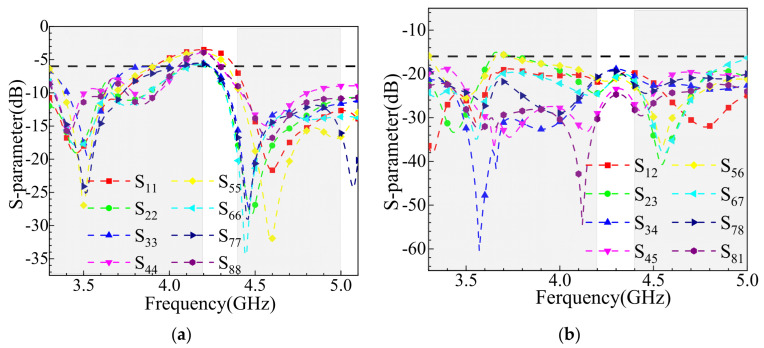
S-parameter simulation results for two-handed use mode: (**a**) reflection coefficient; (**b**) coupling.

As shown in Figure 17b, the dual-hand mode model of the MIMO antenna demonstrates a different obstruction pattern compared to the single-hand mode. In the dual-hand mode, Ant.1, Ant.4, Ant.5, and Ant.8 are primarily obstructed, while the remaining antenna elements remain unobstructed. Figure 20a reveals that in the dual-hand mode, the MIMO antenna’s reflection coefficients remain below −6 dB, with the bandwidth still covering the 3.3–3.6 GHz and 4.8–5.0 GHz frequency ranges. Figure 20b indicates that the isolation performance remains strong, with isolation levels consistently above 16 dB across the operating frequency range where the reflection coefficient is below −6 dB. This demonstrates the antenna’s robustness and reliable performance even under dual-hand usage conditions.

**Figure 21:**
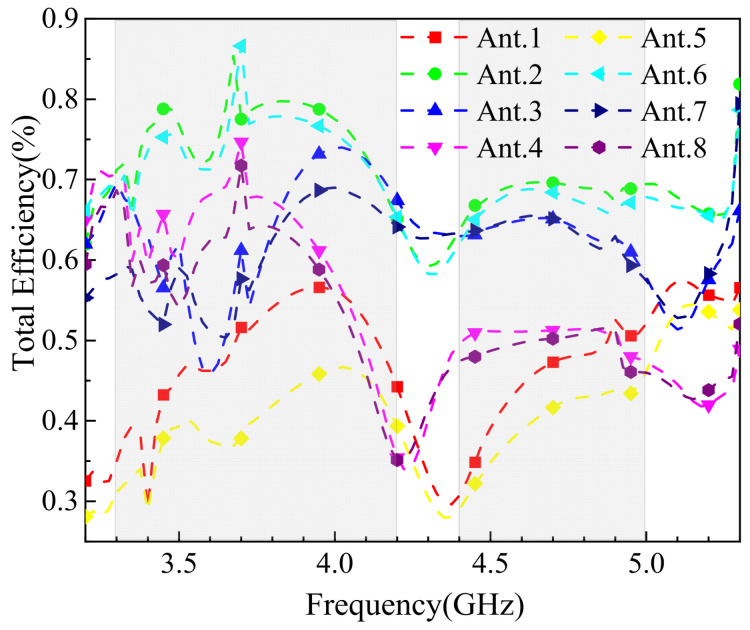
Simulation results of antenna efficiency for two-handed use mode.

Figure 21 presents the simulated radiation efficiency of the antenna in dual-hand usage mode. The radiation efficiency of Ant.1 and Ant.8 is most significantly affected, dropping to only 30% at 3.5 GHz, similar to the single-hand mode. However, the efficiency of the other antenna elements remains largely unaffected, maintaining values above 40% within the 3.3–3.6 GHz and 4.8–5.0 GHz ranges. This meets industry standards and ensures the MIMO antenna’s normal operation, demonstrating its robustness in dual-hand usage scenarios.

## 4. Experimental Validation and Comparative Analysis

**Figure 22:**
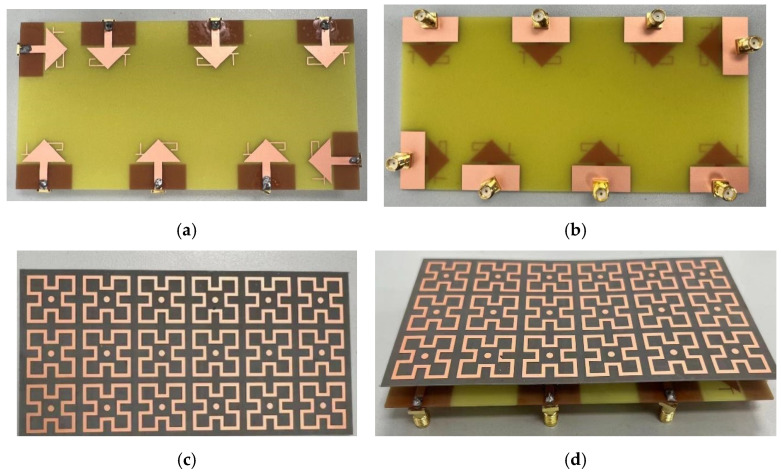
Prototype of the designed antenna array and metasurface: (**a**) top view of the antenna array; (**b**) bottom view of the antenna array; (**c**) top view of the metasurface; (**d**) stereoscopic view.

To validate the performance of the designed antenna, the proposed 8-element MIMO antenna was fabricated and tested, as shown in Figure 22. The 8-element monopole antenna is printed on a 0.8 mm thick FR4 substrate (Figure 22a), with the ground plane on the reverse side (Figure 22b). The antenna elements are connected to 50 Ω SMA connectors as feeding ports and linked to the ground plane. The metasurface is printed on a 0.5 mm thick F4b substrate (Figure 22c) and placed 9 mm above the antenna (Figure 22d). During testing, a vector network analyzer was used to measure the reflection coefficients, coupling levels, and radiation patterns. When testing one or more ports of the MIMO antenna, all the remaining feeding ports were terminated with 50 Ω loads.

**Figure 23:**
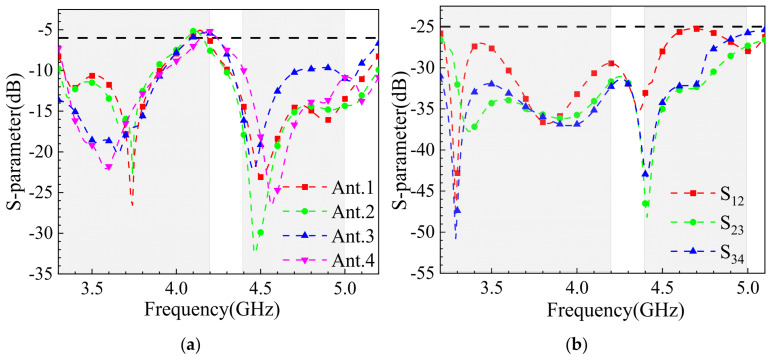
S-parameter measurement results: (**a**) Reflection coefficient; (**b**) coupling.

The measured reflection coefficients and coupling levels of the 8-element MIMO antenna are shown in Figure 23. Due to the symmetrical distribution of the 8 antenna elements on both sides of the model, only the parameters for Ant.1 to Ant.4 are provided. Comparing the measured results with the simulation results (Figure 23a,b), discrepancies are observed due to fabrication and measurement errors. The measured frequency bands cover 3.3–4.08 GHz and 4.25–5.2 GHz, with the lower band narrowing from 3.3–4.17 GHz (simulated) to 3.3–4.08 GHz (measured). Despite this, the design objectives are still met. In terms of isolation, the measured results exceed 25 dB (Figure 23b), outperforming the simulated result of 18 dB (Figure 23b). Overall, the physical testing confirms that the proposed 8-element MIMO antenna effectively covers the 3.3–3.6 GHz and 4.8–5.0 GHz bands while demonstrating excellent isolation performance.

**Figure 24:**
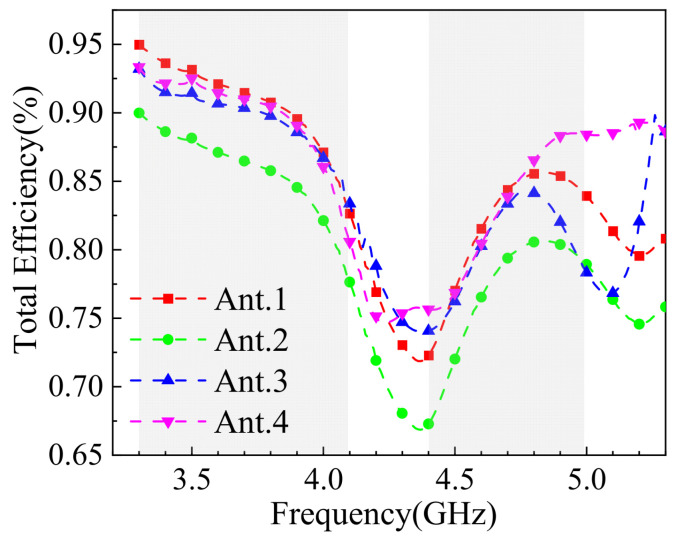
Antenna efficiency simulation results.

Figure 24 presents the radiation efficiency of the 8-element MIMO antenna. As shown in the figure, the overall efficiency exceeds 65%, with noticeable fluctuations around 4.4 GHz, where the minimum efficiency is approximately 67%. Within the 5G frequency bands, the efficiency ranges between 85% and 95% at 3.3–3.6 GHz, while in the 4.8–5.0 GHz band, the efficiency varies more significantly but remains within 65% to 95%. Typically, mobile device antennas are required to achieve an efficiency of at least 40%. The results demonstrate that the designed 8-element MIMO antenna meets and exceeds the general efficiency requirements for mobile device antennas in the industry.

**Figure 25:**
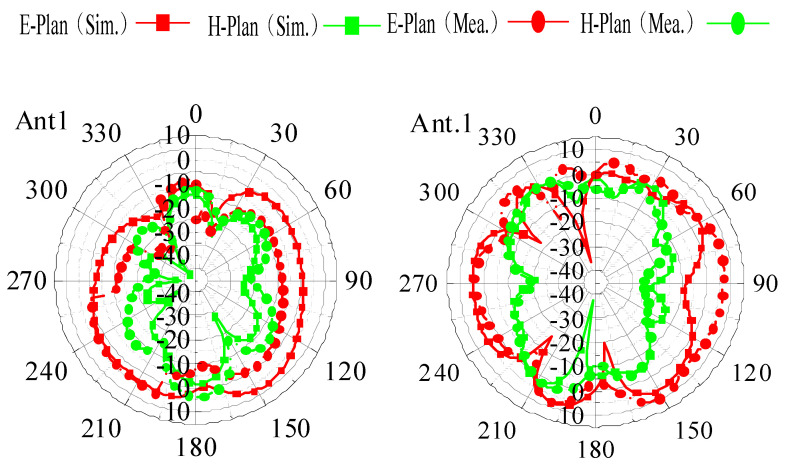

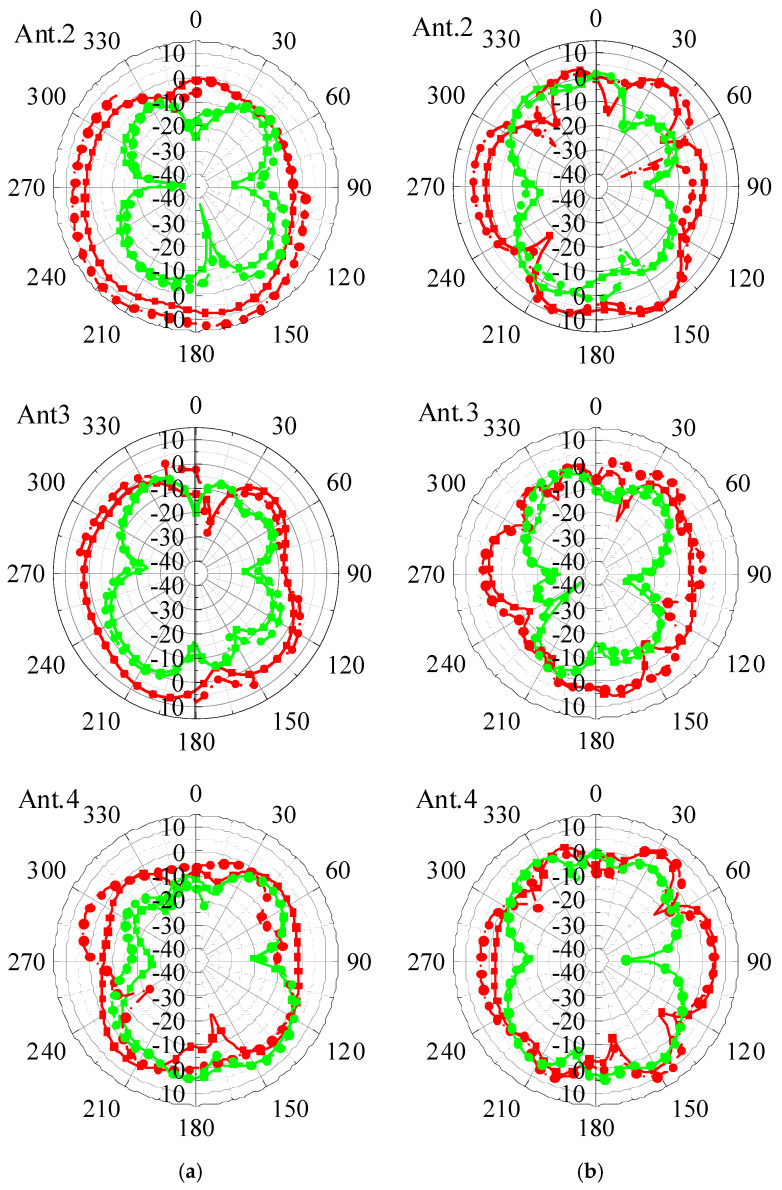
Measured and simulated orientation chart: (**a**) 3.5 GHz; (**b**) 4.9 GHz.

Figure 25a,b present the simulated and measured radiation patterns of Ant.1 to Ant.4 in the 8-element MIMO antenna at 3.5 GHz and 4.9 GHz, respectively. At 3.5 GHz, Ant.1 and Ant.2 exhibit relatively uniform radiation patterns in the E-plane within the range of 30° to 330°, while Ant.3 and Ant.4 show similar patterns in the E-plane but with attenuation in the 0° to 30° range for Ant.3. In the H-plane, all four antenna elements radiate omnidirectionally, with attenuation observed at 0°, 90°, 180°, and 270°, but otherwise consistent patterns. At 4.9 GHz, the radiation patterns are similar to those at 3.5 GHz, with differences primarily in radiation intensity. Discrepancies between the simulated and measured results are likely due to fabrication tolerances and testing environment influences. The radiation patterns confirm that the antenna maintains stable directional performance without significant angular deviations, ensuring reliable operation in mobile devices and minimizing interference during use. Additionally, the peak antenna gain reaches 12 dBi at 3.5 GHz, demonstrating strong radiation performance.

## 5. Conclusions

This paper proposes an eight-element monopole dual-band MIMO antenna designed for 5G communication in mobile devices. Initially, the monopole antenna element was studied and designed, achieving dual-band operation and miniaturization through slotting and branch-loading techniques. The spatial arrangement of the MIMO antenna was then optimized based on its radiation characteristics, with corner elements arranged orthogonally to reduce coupling and maximize space utilization, achieving a baseline isolation of over 16 dB. To further enhance decoupling, a metasurface structure was introduced, improving isolation while increasing gain. To ensure the reliability of the simulation results, a physical prototype was fabricated and tested. The performance parameters of the MIMO antenna were calculated and compared with those of recently reported antennas. Finally, the antenna’s performance under practical handheld scenarios was simulated and analyzed. Overall, the designed MIMO antenna achieves a reflection coefficient below −6 dB in the frequency bands of 3.23–4.14 GHz and 4.31–5.3 GHz, covering the primary 5G communication bands for mobile devices in China (3.3–3.6 GHz and 4.8–5.0 GHz). The antenna exhibits a gain range of 6–12 dBi, isolation exceeding 18 dB, ECC values below 0.05 for all ports, a diversity gain (DG) of 10 dB, a total active reflection coefficient (TARC) below −24 dB, a channel capacity of 39–45 bps/Hz, and a total efficiency above 65%. These results demonstrate the antenna’s excellent performance and suitability for 5G mobile applications.

## Figures and Tables

**Table 1:** Key parameters and corresponding values of the designed MIMO antenna (unit: mm).

Parameters	Value	Parameters	Value	Parameters	Value	Parameters	Value
W1	25	W6	3	L5	3.5	S1	23.5
W2	10.75	L1	11	L6	10	S2	17
W3	3.5	L2	12.5	L7	1.5	S3	39
W4	3.3	L3	0.5	L8	0.5		
W5	20	L4	5	L9	3.5		

**Table 2:** Performance comparison of the proposed 8-cell MIMO antenna with Ref.

Reference	Isolation (dB)	Bandwidth (GHz)	ECC	Efficiency (%)	Dimension (mm^3^)
[15]	>16	3.4–3.6	<0.05	59	340.8
[16]	>10	3.3–4.2	0.1	64	450
[17]	>15	3.3–4.0	0.02	92	304.7
[18]	>15	3.4–3.6	0.02	70	252
This work	>18	3.3–4.0 4.1–5.3	0.05	70	160

## Data Availability

The original contributions presented in this study are included in the article. Further inquiries can be directed to the corresponding author.

## References

[1] Agiwal M., Kwon H., Park S., Jin H. (2021). A survey on 4G-5G dual connectivity: Road to 5G implementation. IEEE Access.

[2] Suthar P., Agarwal V., Shetty R.S., Jangam A. (2020). Migration and Interworking between 4G and 5G. Proceedings of the 2020 IEEE 3rd 5G World Forum (5GWF).

[3] Sarkar D., Srivastava K.V. (2017). Compact four-element SRR-loaded dual-band MIMO antenna for WLAN/WiMAX/WiFi/4G-LTE and 5G applications. Electron. Lett..

[4] Sun L., Li Y., Zhang Z., Feng Z. (2020). Wideband 5G MIMO Antenna With Integrated Orthogonal-Mode Dual Antenna Pairs for Metal-Rimmed Smartphones. IEEE Trans. Antennas Propag..

[5] Chang L., Yu Y., Wei K., Wang H. (2019). Polarization-Orthogonal Co-frequency Dual Antenna Pair Suitable for 5G MIMO Smartphone With Metallic Bezels. IEEE Trans. Antennas Propag..

[6] Xu H., Gao S.S., Zhou H., Wang H., Cheng Y. (2019). A Highly Integrated MIMO Antenna Unit: Differential/Common Mode Design. IEEE Trans. Antennas Propag..

[7] Chang L., Yu Y., Wei K., Wang H. (2020). Orthogonally Polarized Dual Antenna Pair With High Isolation and Balanced High Performance for 5G MIMO Smartphone. IEEE Trans. Antennas Propag..

[8] Li M.Y., Ban Y.L., Xu Z.Q., Wu G., Kang K., Yu Z.F. (2016). Eight-Port Orthogonally Dual-Polarized Antenna Array for 5G Smart-phone Applications. IEEE Trans. Antennas Propag..

[9] Deng C., Liu D., Lv X. (2019). Tightly Arranged Four-Element MIMO Antennas for 5G Mobile Terminals. IEEE Trans. Trans. Trans. Trans. Trans. Trans. Antennas Propag..

[10] Sun L., Li Y., Zhang Z. (2021). Wideband Integrated Quad-Element MIMO Antennas Based on Complementary Antenna Pairs for 5G Smartphones. IEEE Trans. Antennas Propag..

[11] Chen H.D., Tsai Y.C., Kuo C. (2020). Broadband Eight-Antenna Array Design for Sub-6 GHz 5G NR Bands Metal-Frame Smartphone Applications. IEEE Antennas Wirel. Propag. Lett..

[12] Li Y., Luo Y., Yang G. (2019). High-isolation 3.5 GHz eight-antenna MIMO array using balanced open-slot antenna element for 5G smartphones. IEEE Trans. Antennas Propag..

[13] Su S.W., Lee C.T., Chang F.S. (2011). Printed MIMO-antenna system using neutralization-line technique for wireless USB-dongle applications. IEEE Trans. Antennas Propag..

[14] Ren Z., Zhao A., Wu S. (2019). MIMO Antenna With Compact Decoupled Antenna Pairs for 5G Mobile Terminals. IEEE Antennas Wirel. Propag. Lett..

[15] Ren A., Liu Y. (2019). A compact building block with two shared-aperture antennas for eight-antenna MIMO array in metal-rimmed smartphone. IEEE Trans. Antennas Propag..

[16] Xu Z., Deng C. (2020). High-isolated MIMO antenna design based on pattern diversity for 5G mobile terminals. IEEE Antennas Wirel. Propag. Lett..

[17] Wong K.L., Jian M.F., Chen C.J., Chen J.Z. (2021). Two-Port Same-Polarized Patch Antenna Based on Two Out of-Phase TM10 Modes for Access-Point MIMO Antenna Application. IEEE Antennas Wirel. Propag. Lett..

[18] Wong K.L., Chen Y.H., Li W.Y. (2018). Decoupled compact ultra-wideband MIMO antennas covering 3300~6000 MHz for the fifth-generation mobile and 5GHz-WLAN operations in the future smartphone. Microw. Opt. Technol. Lett..

[19] Zhao A., Ren Z. (2019). Wideband MIMO antenna systems based on coupled-loop antenna for 5G N77/N78/N79 applications in mobile terminals. IEEE Access.

[20] Al-Bawri S.S., Islam M.T., Singh M.J., Alyan E., Jusoh M., Sabapathy T., Hossain K. (2021). Broadband Sub-6GHz Slot-based MIMO antenna for 5G NR bands mobile applications. J. Phys. Conf. Ser..

